# Supplementary material for: Probing active sites for carbon oxides hydrogenation on Cu/TiO2 using infrared spectroscopy
Source: Commun Chem. 2022 Mar 14;5:32. doi: 10.1038/s42004-022-00650-2 (PMC9814513; doi:10.1038/s42004-022-00650-2)
Supplement: Supplementary file 1 — Supplementary Information [file 42004_2022_650_MOESM1_ESM.pdf]

Supplementary Information for

Probing Active Sites for Carbon Oxides Hydrogenation on Cu/TiO_2 Using Infrared Spectroscopy

*Ehab Shaaban and Gonghu Li **

Department of Chemistry, University of New Hampshire, Durham, NH 03824, United States

* To whom correspondence should be addressed. Email: gonghu.li@unh.edu

Supplementary Figures

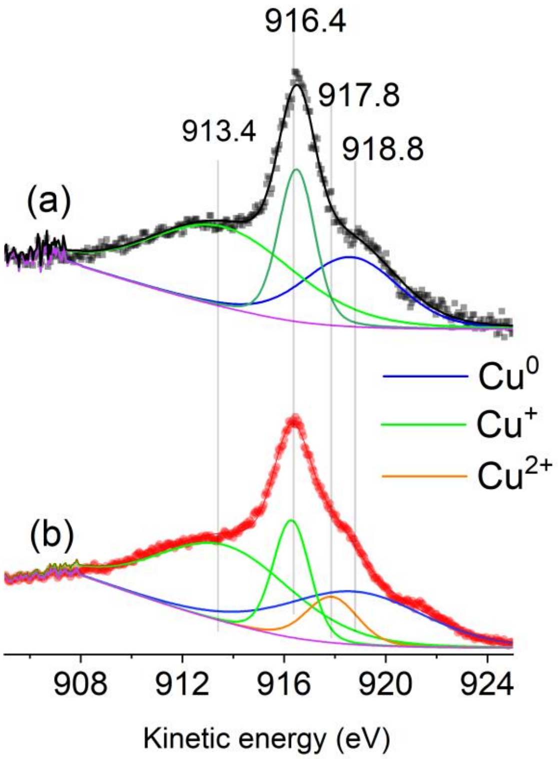

Figure S1. Fitting Cu LMM X-ray-excited Auger electron spectra of (a) Cu/TiO_2-H_2 after the catalyst was pretreated at 300 ℃ under hydrogen for 1 hr; (b) Cu/TiO_2-H_2-Ar after the hydrogen-treated sample was further treated at 300 ℃ under Ar. Reference [1] was used to guide this fitting.

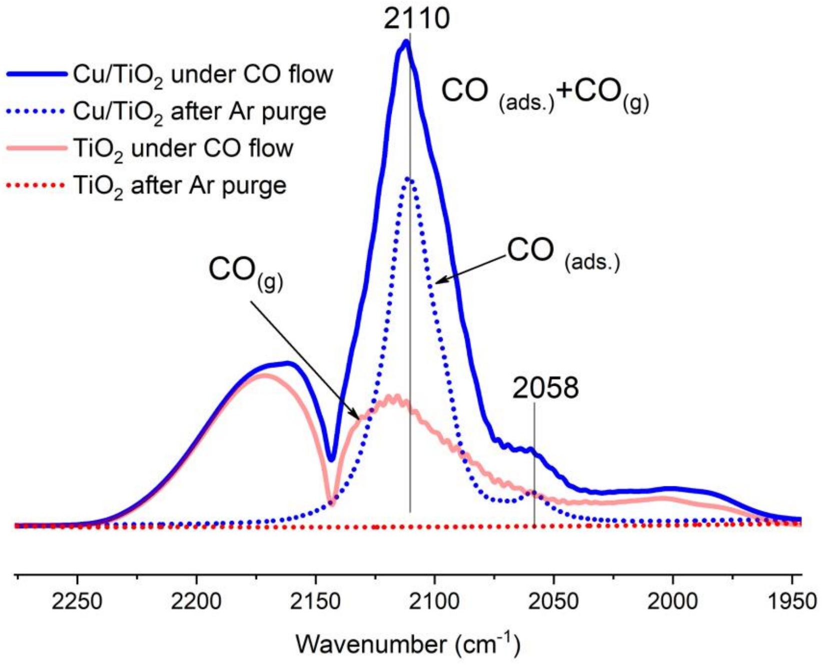

Figure S2. DRIFTS spectra of Cu/TiO_2 (blue) and TiO_2 (red) under CO flow for 5 minutes (solid lines) then after Ar purge for 1 minute (dotted lines).

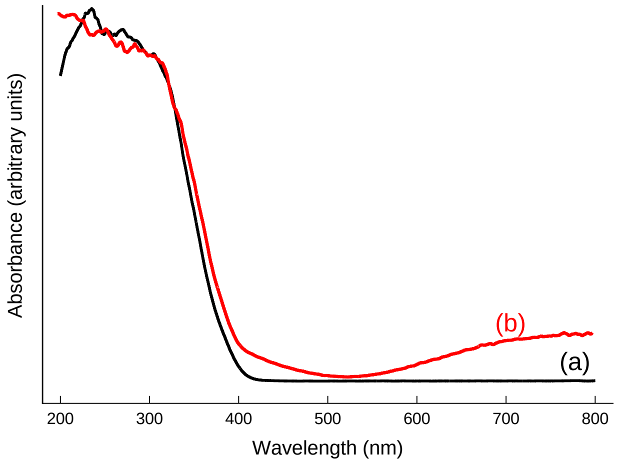

Figure S3. Diffuse reflectance UV-vis spectra of (a) TiO_2 and (b) Cu/TiO_2.

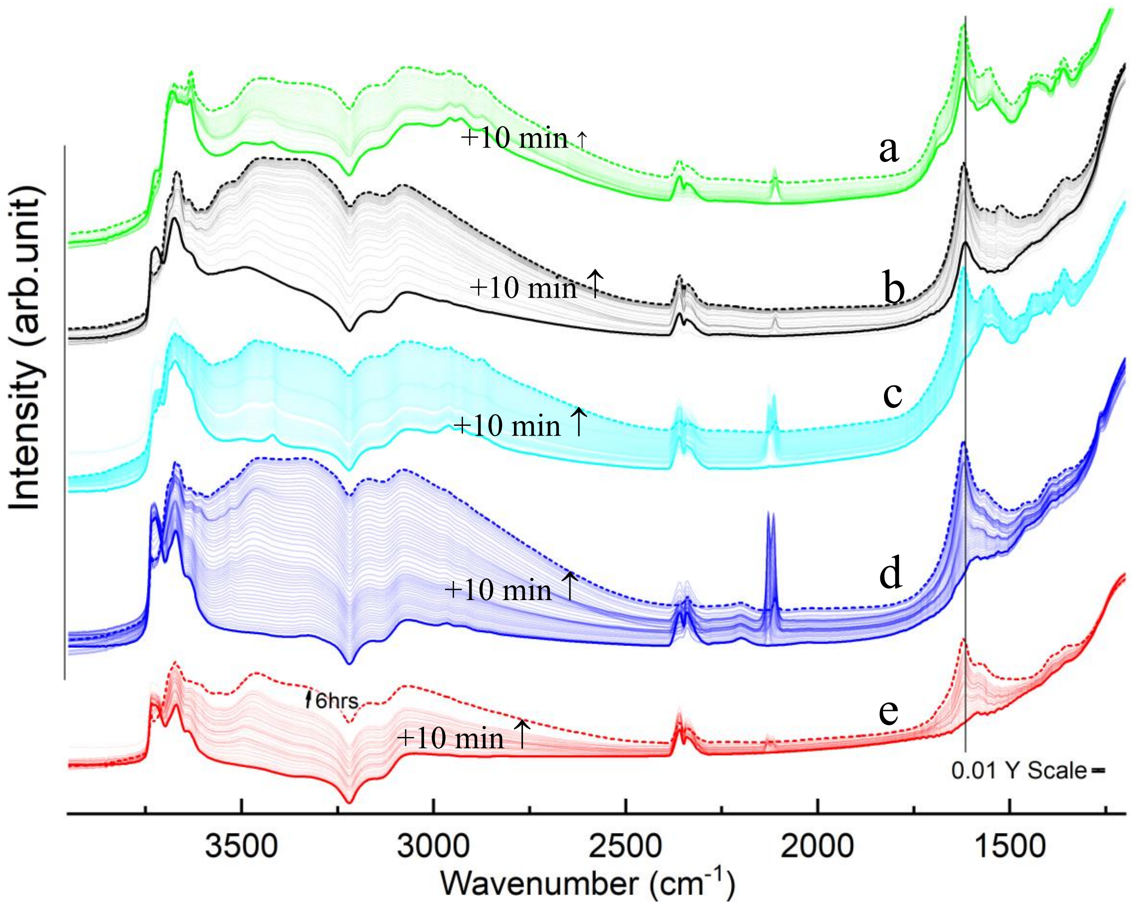

Figure S4. Full DRIFTS spectra for $CuTiO_2$ with time at RT after pretreatment at 100 ℃ (a), 150 ℃ (b), 200 ℃ (c), 300 ℃ (d), and 400 ℃ (e). Solid lines are collected when the sample reached RT after activation and the dotted lines are collected at the end of the measurement (water vapor traces that diffuse from the cell walls are re-adsorbed). The dip around 3200 cm^{-1} is an artifact due to spectral subtraction.

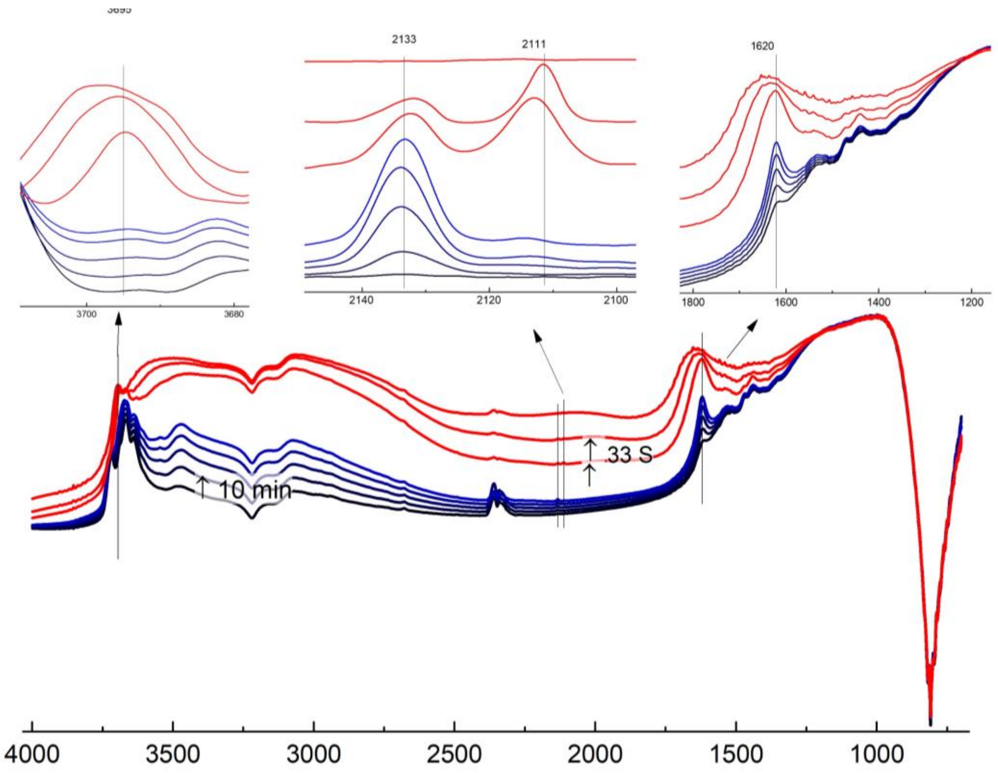

Figure S5. Full spectra for Figure 5 that represent Cu/TiO$_2$ *before (black to blue, collected every 10 minutes) and after (red, collected every 33 seconds) the introduction of water vapor into the IR cell. Water vapor was purposely introduced in the middle of the rise of the HF CO peak.*

Reference

1. Biesinger, M. C. Advanced analysis of copper X-ray photoelectron spectra. *Surf. Interface Anal.* **49**, 1325–1334 (2017).
